# Super-large record-breaking mitochondrial genome of *Cathaya argyrophylla* in Pinaceae

**DOI:** 10.3389/fpls.2025.1556332

**Published:** 2025-06-19

**Authors:** Kerui Huang, Wenbo Xu, Haoliang Hu, Xiaolong Jiang, Lei Sun, Wenyan Zhao, Binbin Long, Shaogang Fan, Zhibo Zhou, Ping Mo, Xiaocheng Jiang, Jianhong Tian, Aihua Deng, Peng Xie, Yun Wang

**Affiliations:** ^1^ Key Laboratory of Agricultural Products Processing and Food Safety in Hunan Higher Education, Science and Technology Innovation Team for Efficient Agricultural Production and Deep Processing at General University in Hunan Province, Hunan University of Arts and Science, Changde, China; ^2^ School of Traditional Chinese Pharmacy, China Pharmaceutical University, Nanjing, China; ^3^ College of Forestry, Central South University of Forestry and Technology, Changsha, China; ^4^ Key Laboratory of Research and Utilization of Ethnomedicinal Plant Resources of Hunan Province, College of Biological and Food Engineering, Huaihua University, Huaihua, China; ^5^ College of Life Sciences, Hunan Normal University, Changsha, China

**Keywords:** *Cathaya argyrophylla*, super-large mitochondrial genome, Pinaceae, RNA-editing, MTPTs

## Abstract

**Introduction:**

Mitochondrial genomes (mitogenomes) in Pinaceae are notable for their large size and complexity. This study investigates the mitogenome of the critically endangered *Cathaya argyrophylla* to understand the drivers of its exceptional genome expansion.

**Methods:**

We sequenced, assembled, and annotated the *C. argyrophylla* mitogenome. Comparative analyses were performed against other Pinaceae species and gymnosperms, examining repeat sequences, transposable elements (LINEs, LTRs), RNA editing events, chloroplast-derived sequence transfers (mtpts), and nuclear genome homology.

**Results:**

The *C. argyrophylla* mitogenome is a record-breaking 18.99 Mb. While *C. argyrophylla* and other extremely large Pinaceae mitogenomes possess substantial repeats and elevated transposon activity, these factors alone do not explain their size. Significant incorporation of mtpts was observed. Additionally, large mitogenomes exhibited distinct RNA editing patterns and reduced nuclear homology compared to smaller genomes.

**Discussion:**

Massive Pinaceae mitogenomes are characterized by a combination of features: substantial repeat content, elevated transposon activity, extensive plastid sequence integration, and distinct RNA editing and nuclear homology patterns. This comprehensive analysis enhances our understanding of plant mitogenome evolution and provides a genomic foundation for *C. argyrophylla* conservation and potential applications.

## 1 Introduction

Mitochondria play a crucial role in energy metabolism within plant cells, and their genomes are essential for studies on plant evolution, physiological functions, and genetic diversity (Edera et al., 2018; Kan et al., 2020; Mower, 2020; Small et al., 2020; Wu et al., 2015; Zhang et al., 2023). While the mitochondrial genomes (mitogenomes) of animals and fungi are relatively simple in structure, plant mitogenomes are known for their significant variation in size, structural complexity, and frequent recombination. Specifically, plant mitogenomes vary dramatically in size, ranging from extremely small to enormous (Wang et al., 2024; Wu et al., 2020; Yu et al., 2021). Structurally, they are highly diverse, existing in circular, linear, and multi-chromosomal conformations, and exhibiting dynamic changes (Qu et al., 2023; Bi et al., 2024). Further, frequent genome rearrangements mediated by repetitive sequences occur, which can integrate large amounts of foreign DNA, further increasing their complexity (Choi and Park, 2021; Smith and Keeling, 2015; Wu and Sloan, 2019; Yu et al., 2022). Research on plant mitogenomes helps to elucidate plant cell evolution, the origins and differentiation of species, the mechanisms behind genomic structural diversity, as well as the interaction and co-evolution between the mitochondrial and nuclear genomes (Kan et al., 2020; Small et al., 2020; Jackman et al., 2020; Jiang et al., 2023).

In plant mitogenomes, the substantial variation in genome size is particularly striking. The mitogenome of plants ranges from 66 kb in the parasitic plant *Viscum scurruloideum* to 11.7 Mb in the Siberian larch (*Larix sibirica*), with considerable differences even between closely related species (Sloan et al., 2018; Sang et al., 2019; Sullivan et al., 2020; Chevigny et al., 2020; Cheng et al., 2021; Kozik et al., 2019; Putintseva et al., 2020). This provides a unique opportunity to investigate the evolutionary mechanisms underlying plant mitogenome diversity. Among plants with exceptionally large mitogenomes, the Pinaceae family have garnered attention due to the massive mitogenomes reported in several species (Kan et al., 2020; Jackman et al., 2020; Putintseva et al., 2020). For instance, *L. sibirica* possesses the largest known mitogenome at 11.7 Mb (Putintseva et al., 2020), and *Picea sitchensis* also has a notably large mitogenome at 5.5 Mb (Jackman et al., 2020). Although initial reports exist, the commonalities and mechanisms behind the enormous size of Pinaceae mitogenomes remain largely unexplored. Previous studies have proposed that factors such as repeat sequences, transposon activity, RNA editing events, and the transfer of genomic fragments between organelles play significant roles in the growth and complexity of plant mitogenomes (Edera et al., 2018; Kan et al., 2020; Small et al., 2020; Sullivan et al., 2020; Cheng et al., 2021; Wynn and Christensen, 2019; Putintseva et al., 2020), For example, repeat sequences account for over 42.5% of the total length of the *Avena longiglumis* mitogenome (Liu et al., 2023), and various types of transposable elements, accounting for approximately 11%, have been found in the *Larix sibirica* mitogenome (Putintseva et al., 2020). However, the shared mechanisms underlying these factors and their potential contributions to the expansion of Pinaceae mitogenome sizes remain largely unclear. Additionally, the extent to which genomic exchanges among the mitogenome, nuclear genome, and chloroplast genome contribute to the large mitogenome sizes in Pinaceae species is a question that demands further investigation.

*Cathaya argyrophylla*, as an ancient member of the Pinaceae family and endemic to China, is renowned for its extreme endangerment and rarity, earning the nickname “Panda of the plant kingdom” due to its special evolutionary status and research value (Huang et al., 2022; Xie et al., 2023; Wang and Ge, 2006). In this study, we sequenced and assembled the mitogenome, revealing that, similar to other Pinaceae species with large mitogenomes, *C. argyrophylla* also possesses an exceptionally large genome at 18.99 Mb, which is the largest mitogenome reported to date, breaking previous records. Why do Pinaceae species, including *C. argyrophylla*, possess such exceptionally large mitogenomes? What are the shared characteristics of these expansive genomes, and how do they differ from or relate to the smaller mitogenomes found in other Pinaceae species and gymnosperms? Answering these questions is essential for gaining deeper insights into the structure, function, and evolution of plant mitogenomes.

To address the aforementioned scientific questions, this study conducted a comprehensive analysis of the mitogenome of *C. argyrophylla*, focusing on several key aspects: the quantity, coverage, patterns of repeat sequences; the patterns of transposable elements, particularly long interspersed nuclear elements (LINEs), long terminal repeats (LTR) elements; the number and patterns of RNA editing events; the quantity, length, and coverage of chloroplast-derived fragments (mtpts); and the sequence homology between the mitogenome and the nuclear genome. A systematic comparison was also performed between *C. argyrophylla* and other Pinaceae species with large mitogenomes, as well as species with smaller mitogenomes (for detailed definitions of large and small genomes, refer to the Materials and Methods section), along with other gymnosperms, to uncover common features and potential mechanisms underlying the formation of large mitogenomes. Through this study, we aim to elucidate the uniqueness of large Pinaceae mitogenomes and explore the relationship between genome size and factors such as repeat sequences, transposable element activity, and organelle-to-organelle sequence exchange. Our findings will provide new perspectives and evidence for understanding the characteristics and evolution of plant mitogenomes. Furthermore, the in-depth study of *C. argyrophylla* will offer essential genetic foundations for its conservation and potential utilization.

## 2 Materials and Methods

### 2.1 Sampling, DNA & RNA extraction, and sequencing

The fresh leaves of *C. argyrophylla* were collected from Xinning County, Hunan Province (26.433 N, 110.847 E) and subsequently deposited at the Key Laboratory of Agricultural Products Processing and Food Safety at Hunan University, with the voucher specimen labeled as CA01. Genomic DNA was extracted from powdered *C. argyrophylla* leaves using the CTAB method, while RNA extraction was performed using the BioTeke Kit (RP3301). The purified DNA and RNA samples, which demonstrated both high concentration and quality as assessed by spectrophotometry and gel electrophoresis, were then sent to Wuhan Benagene Technology Co., Ltd. for high-throughput sequencing. For long-read sequencing, libraries were constructed using the Oxford Nanopore Technologies (ONT) Ligation Sequencing Kit (SQK-LSK110). Meanwhile, short-read sequencing libraries were prepared using the Plus DNA Library Prep Kit (MGI, NDM627) and sequenced on the DNBSEQ-T7 platform. For short-read data, low-quality reads and adapter sequences were removed using Trimmomatic with default settings. For long-read data, quality control was conducted using NanoComp to filter out low-quality sequences from the raw output.

### 2.2 Genomes assembly and annotation

The contigs of *C. argyrophylla* were first assembled using Flye with ONT sequencing reads (Kolmogorov et al., 2019). The coding regions of *Pinus taeda* (NC037304) and *Liriodendron tulipifera* (NC021152) were used as seed to select mitogenome sequences (Edera et al., 2018). Subsequently, the coding sequences of *Pinus taeda* and *Liriodendron tulipifera* were used as queries against the Flye-assembled contigs (as subjects) using BLASTn with default parameters to find matching sequences. SeqKit was then used to extract these aligned contigs. Furthermore, as the algorithm description for mapping ONT reads with BWA (Li and Durbin, 2009) was ambiguous, Unicycler (Wick et al., 2017) was employed to complete this process. Final assembled genome sequences were performed with Bandage (Wick et al., 2015). To further validate the assembly accuracy of each circular genome, we extracted 40 kb fragments from both the start and end of every contig and ligated these paired fragments with 20 kb N spacers. We then mapped randomly selected original sequencing reads back to the assembled genomes using minimap2. Results from MUMmer visualization demonstrated that reads spanned both the start and end of the sequences, indicating physical continuity between the original termini of each contig, thus confirming the assembly accuracy (**Supplementary Figure S1**). In addition, to further ensure the accuracy of the assembly results, we utilized an additional 200G of *C. argyrophylla* second-generation sequencing data (unpublished data) and mapped these reads to the assembled contigs. We used BWA to index the *C. argyrophylla* mitochondrial genome and align these second-generation sequencing reads. SAMtools (Zhang et al., 2023) was employed to sort the resulting alignment files. Subsequently, the depth at each site was calculated. The results showed that the coverage depth was evenly distributed across the entire mitochondrial assembly, at approximately 45× (**Supplementary Figure S2**). All these analyses collectively indicate that our assembly results are reliable.

PMGA, tRNAscan-SE, and BLASTn (Li et al., 2024; Lowe and Eddy, 1997; Chen et al., 2015) were used to annotate protein-coding genes, transfer RNAs (tRNAs), and ribosomal RNAs (rRNAs) in the mitogenome, respectively. After corrections in Apollo and CPStools, the final annotations were submitted to the NCBI database under accession numbers PP764533 to PP764541 (Lewis et al., 2002; Huang et al., 2024). The plastome genome was assembled and annotated using GetOrganelle and CPGAVAS2 (Jin et al., 2020; Shi et al., 2019). After manual correction, it was deposited in NCBI with the accession number OL790355.

### 2.3 Comparison and classification of gymnosperm mitogenomes

To better explore the unique features of the *C. argyrophylla* mitogenome, 10 other gymnosperm species were selected, including *Abies koreana* (NC071216), *Cycas taitungensis* (AP009381), *Ginkgo biloba* (KM672373), *L. sibirica* (MT797187-MT797195), *P. sitchensis* (MK697696-MK697708), *P.taeda* (MF991879), *Platycladus orientalis* (OL703044-OL703045), *Taxus cuspidat*a (MN593023), *Thuja sutchuenensis* (ON603305-ON603308), and *Welwitschia mirabilis* (KT313400). The selection criteria were based on the availability of mitogenomes in NCBI, aiming to distribute species as evenly as possible across the spectrum of mitogenome sizes. Furthermore, only one species was chosen from the same genus when their mitogenomes were similar in size. These species encompass a wide range of mitogenome sizes, allowing us to classify them as follows: mitogenome sequence lengths less than 2M were categorized as ‘small,’ those between 2M and 5M as ‘large,’ and those 5M or larger as ‘extremely large’.

### 2.4 Synteny analysis

BLASTn, with an default e-value set to 1e-5, was used to identify homologous fragments between the mitogenome of *C. argyrophylla* and those of 10 other gymnosperms mentioned above. Homologous fragments shorter than 200 bp were filtered out. The remaining fragments were then formatted and visualized using MCScanX (Wang et al., 2012), based on their genomic positions.

### 2.5 Repeat sequence analysis

RepeatModeler (Flynn et al., 2020) was used to identify repeat elements in the mitogenome, while the TEclass online service was employed to classify unknown repeat elements from the *de novo* repeat library generated by RepeatModeler. Then the RepeatMasker results based on the RepBase (Jurka et al., 2005) classification were parsed and analyzed.

### 2.6 RNA editing analysis

In *C. argyrophylla*, Illumina transcriptome sequencing of leaf tissue from the same individual enabled the identification of RNA editing events. After aligning the transcriptome data to the assembled mitogenome, Bedtools (Quinlan, 2014) was used to detect these events, retaining only those with a frequency greater than 60%. Data for RNA editing sites in other species were obtained from previously published studies. To ensure data rigor, we utilized only experimentally validated RNA editing data from the literature for comparison, specifically excluding any non-validated or computationally predicted sites. This curated dataset included *Oryza sativa* (Notsu et al., 2002), *Arabidopsis thaliana* (Small et al., 2020; Bentolila et al., 2013), *Welwitschia mirabilis* (Kan et al., 2020), *Liriodendron tulipifera* (Edera et al., 2018), and *Larix sibirica* (Putintseva et al., 2020).

### 2.7 MTPT sequence transfer analysis

BLASTn, with an default e-value set to 1e-5, was used to identify potential homologous sequences transferred between the plastomes and mitogenomes of *C. argyrophylla* and other gymnosperm species, using the plastome of *C. argyrophylla* (OL790355) from our previous study along with plastomes from *A. koreana* (NC026892), *C. taitungensis* (NC009618), *G. biloba* (MN443423), *L. sibirica* (NC036811), *P. sitchensis* (NC011152), *P. taeda* (KY964286), *P. orientalis* (KX832626), *T. cuspidata* (NC041498), *T. sutchuenensis* (NC042176), and *W. mirabilis* (EU342371). The BLAST results were visualized using TBtools (Chen et al., 2020), and the homologous sequences were checked to determine whether they contained complete or partial genes originating from the chloroplast.

### 2.8 NUMTs sequences transfer analysis

This study analyzed the sequences transfer between the mitochondrial and nuclear genomes (NUMTs) in gymnosperms and angiosperms. For angiosperms, the complete nuclear genome sequences of *Arabidopsis thaliana* (GCF_000001735.4), *Asparagus officinalis* (GCF_001876935.1), *Glycine max* (GCF_000004515.6), *Nicotiana tabacum* (GCF_000715135.1), *Oryza sativa* (GCF_034140825.1), and *Zea mays* (GCF_902167145.1) were downloaded for analyzing. The first three species represented dicotyledons, while the latter three represented monocotyledons. For gymnosperms, the complete nuclear genome sequences of *P. taeda* (GCA_000404065.3), *L. sibirica* (GCA_004151065.3), *P. sitchensis* (GCA_010110895.2), and *Cycas panzhihuaensis* (GCA_023213395.1) were downloaded for analyzing.

We used the complete nuclear genome sequences of these species as indexes. Then, BLASTn was employed to align the mitogenomes of Pinaceae species and gymnosperms to these nuclear genome indexes to identify homologous fragments. The BLASTn parameter settings were: E-value = 1e-5, and only fragments longer than 100 bp were retained as NUMTs.

## 3 Results

### 3.1 Genomes assembly and annotation

**Figure 1:**
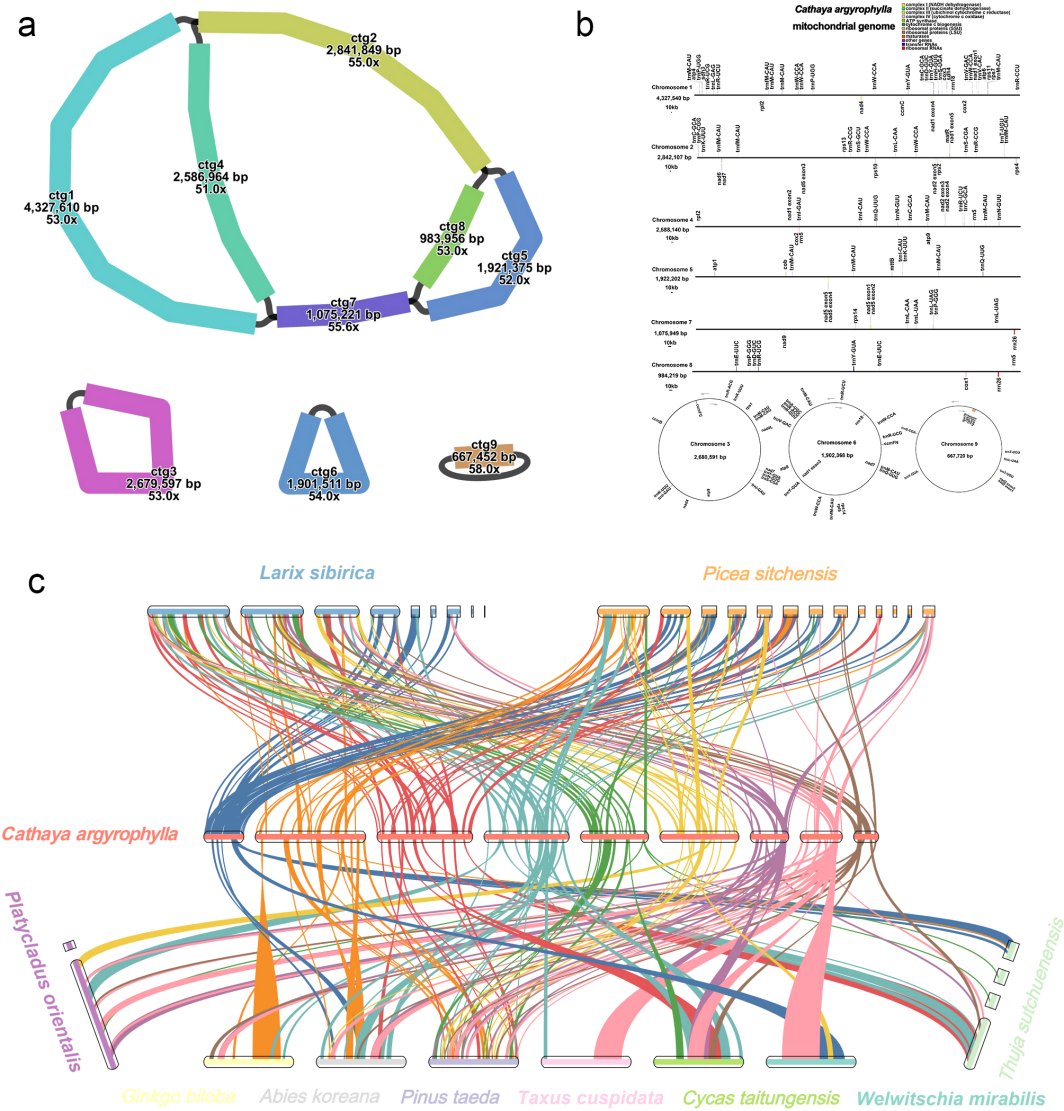
Structure and annotation of the mitochondrial genome of *C. argyrophylla* and its collinearity with those of related species. **(a)** The structure of *C. argyrophylla* mitochondrial genome, illustrating its multi-branched configuration, including six linear contigs and three circular contigs. **(b)** Gene annotation information of *C. argyrophylla*, highlighting protein-coding genes, tRNAs, rRNAs, and other functional elements. **(c)** Synteny analysis between *C. argyrophylla* and other gymnosperms, showing homologous blocks and structural conservation across mitochondrial genomes.

A total of 50 Gb ONT reads and 69.6 Gb Illumina reads were utilized for assembling the mitogenome. The mitogenome displayed a complex multi-branched structure, consisting of three circular chromosomes and six linear chromosomes (**Figure 1a**), with a total length of 18,990,836 bp and a GC content of 44.06%. This genome represents the largest mitogenome reported to date among gymnosperms. A total of 75 genes were annotated in the mitogenome of *C. argyrophylla* (**Figure 1b**), including 40 protein-coding genes (24 core genes and 16 non-core genes), 32 tRNA genes (25 of which are multi-copy), and three rRNA genes (all of which are multi-copy). The core genes included five ATP synthase genes (*atp1*, *atp4*, *atp6*, *atp8*, *atp9*); nine NADH dehydrogenase genes (*nad1*, *nad2*, *nad3*, *nad4*, *nad4L*, *nad5*, *nad6*, *nad7*, *nad9*); four cytochrome c biogenesis genes (*ccmB*, *ccmC*, *ccmFC*, *ccmFN*); three cytochrome c oxidase genes (*cox1*, *cox2*, *cox3*); one membrane transporter gene (*mttB*); one maturase gene (*matR*); and one ubiquinol-cytochrome c reductase gene (*cob*). The non-core genes comprised three large ribosomal subunit genes (*rpl2*, *rpl5*, *rpl16*); 11 small ribosomal subunit genes (*rps1*, *rps2*, *rps3*, *rps4*, *rps7*, *rps10*, *rps11*, *rps12*, *rps13*, *rps14*, *rps19*); and two succinate dehydrogenase genes (*sdh3*, *sdh4*).

### 3.2 Comparative synteny analysis among Pinaceae mitogenomes

The homology results show that *C. argyrophylla* shares numerous syntenic blocks with Pinaceae species, often exhibiting significant rearrangements (**Figure 1c**). Additionally, *C. argyrophylla* has more syntenic blocks with species that have larger mitogenomes (**Figure 1c**), though the number of these blocks decreases as phylogenetic distance grows. For instance*, L. sibirica* has the second-largest mitochondrial genome among currently published plant mitogenomes, following *C. argyrophylla*, and shares 83 syntenic blocks with *C. argyrophylla*. In contrast, *P. sitchensis*, a closer relative with a mitogenome almost half the size of *L. sibirica*, shares 84 syntenic blocks. Similarly, *P. taeda* and *A. koreana* have similarly small mitogenomes, yet *P. taeda*, being more closely related to *C. argyrophylla*, shares 43 syntenic blocks, while *A. koreana*, which is more distantly related, shares only 18 blocks (**Figure 1c**). For non-Pinaceae species, the number of syntenic blocks shared with *C. argyrophylla* is significantly lower, all at or below 10. These findings suggest that closer phylogenetic relationships correspond to greater mitogenome sequence homology, reinforcing the relatedness among Pinaceae species. Furthermore, within Pinaceae, species with larger mitogenomes tend to share more syntenic blocks, indicating that mitogenome size may influence syntenic relationships within the family. However, the extent to which mitogenome size influences the number of syntenic blocks in other taxonomic groups warrants further investigation.

### 3.3 Relationship between repeat sequences and mitogenome size in Pinaceae

**Figure 2:**
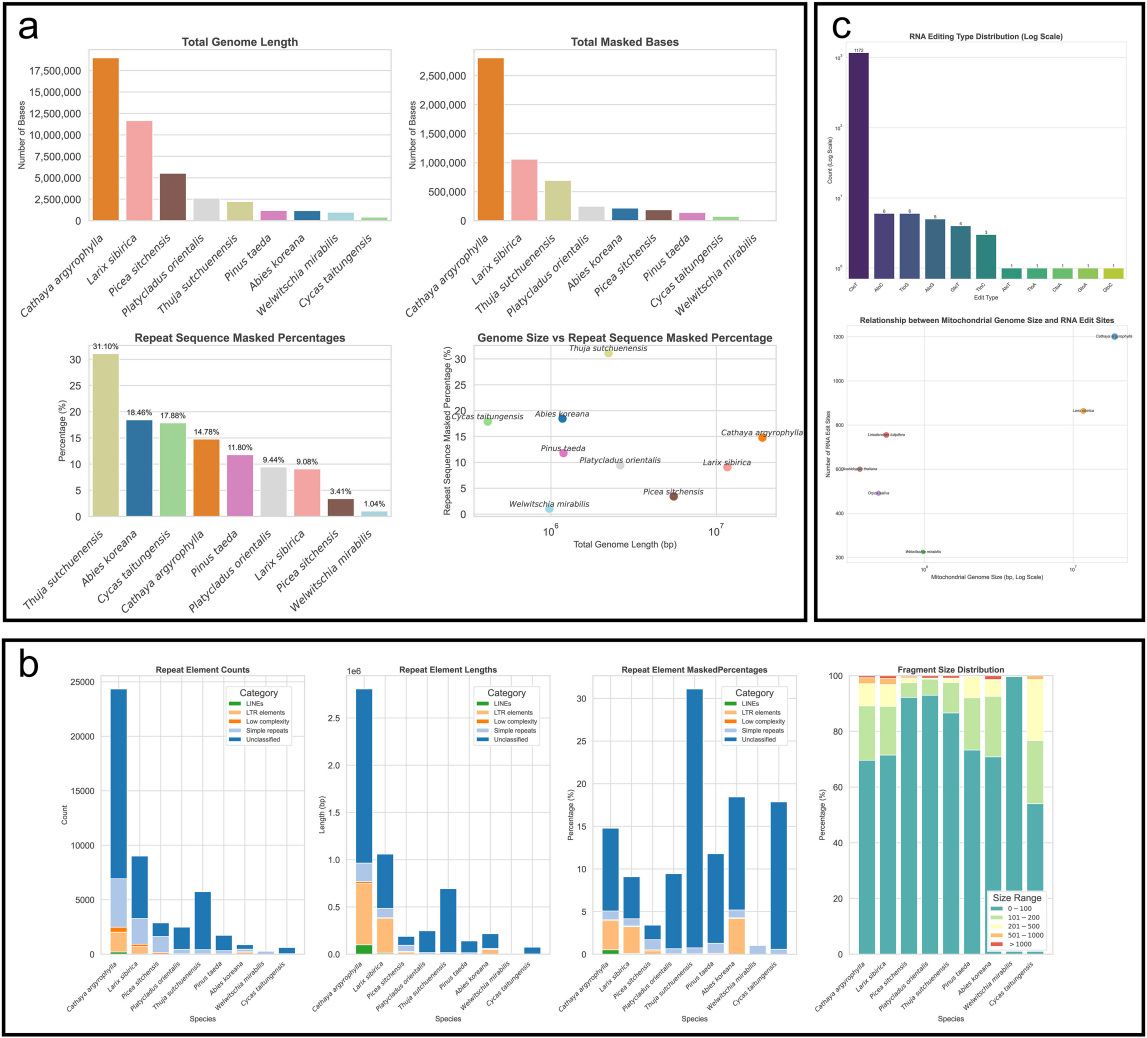
Comparative analysis of repeat sequences and RNA editing between *C. argyrophylla* and other gymnosperms or angiosperms. **(a)** Comparative analysis of repeat sequences between *C. argyrophylla* and other gymnosperms. **(b)** Comparison of repeat sequence types between *C. argyrophylla* and other Pinaceae species. **(c)** Comparative analysis of RNA editing sites between *C. argyrophylla* and various other plants. The RNA editing sites for *C. argyrophylla* were determined using second-generation sequencing data from this study. The RNA editing data for the other species used for comparison were all from previous studies with experimentally verified data, detailed in the Materials and Methods section.

Analysis of repeat sequences shows that *C. argyrophylla*, similar to *L. sibirica*, which also has an extremely large mitogenome, possesses the longest total length of repeat sequences, as well as the longest total length of its genome covered (masked) by these repeats (**Figure 2a**, **b**). However, the proportion of repeat sequences in the *C. argyrophylla* mitogenome is not the highest, accounting for 14.78%, while *L. sibirica* exhibits an even lower proportion at 9.08%. In contrast, small mitogenomes, such as those of *T. sutchuenensis* (31.10%), *A. koreana* (18.46%), and *C. taitungensis* (17.88%), exhibit the higher repeat sequence coverage, all surpassing that of *C. argyrophylla* and *L. sibirica*. This suggests that large mitogenomes are not predominantly composed of repeat sequences. Interestingly, in species with extremely large mitogenomes (such as *C. argyrophylla*, *L. sibirica*, and *P. sitchensis*), the coverage of repeat sequences increases with genome size, indicating that among extremely large mitogenomes, larger mitogenomes are more prone to contain repeat sequences. This pattern is not observed in other mitogenomes, where some of the smallest genomes show the highest repeat sequence coverage, highlighting significant differences in sequence composition between extremely large and smaller mitogenomes.

In terms of repeat sequence composition, only five types were identified in the mitogenomes of all species: LINEs, LTR elements, low-complexity sequences, simple repeats, and unclassified sequences (**Figure 2b**). Among these, unclassified sequences were the most common, while the autonomous retrotransposon LINEs were the rarest, and entirely absent in smaller mitogenomes. However, in extremely large genomes, LINEs began to appear in small quantities in *L. sibirica*, and their proportion relative to the total genome length was higher in *C. argyrophylla*, which possesses the largest mitogenome. This observation indicates a higher representation of LINE sequences in the larger mitogenomes within the extremely large category. A similar trend was observed for another autonomous retrotransposon, LTR elements, which, though generally rare, increased in proportion as mitogenome size grew among species with extremely large mitogenomes. This pattern might reflect a greater historical accumulation or retention of these elements, correlating with genome size expansion. Indicating that among extremely large mitogenomes, larger mitogenomes may exhibit higher transposon activity, a phenomenon not observed in smaller genomes, and extremely large mitogenomes may have experienced more frequent high-activity events involving LINEs and LTR elements, and that genome size is likely related to this activity. However, an exception was found in *A. koreana*, a species with a small mitogenome, where the proportion of LTR elements was unusually high, even surpassing that of *C. argyrophylla*. This further highlights the differences in the patterns and mechanisms of repeat sequence formation between small and large mitogenomes.

Further analysis of the relationship between repeat sequence fragment size and mitogenome size revealed that, across all species, the majority of repeat sequences were small fragments between 1–100 base pairs (bp) with their abundance decreasing as fragment size increased (**Figure 2b**). However, in species with extremely large mitogenomes (such as *C. argyrophylla*, *Larix sibirica*, and *Picea sitchensis*), the proportion of repeat fragments longer than 200 bp increased with genome size. While in smaller genomes, this trend was not observed, and in some cases, even showed the opposite pattern. This highlights a clear architectural difference in the composition of repeat sequences between extremely large Pinaceae mitogenomes and the smaller mitogenomes of Pinaceae and other gymnosperms. It also suggests a distinct correlation between genome size and repeat sequence structure in extremely large mitogenomes.

### 3.4 Relationship between RNA editing and genome size in Pinaceae

Next, we evaluated RNA editing events in *C. argyrophylla* using second-generation sequencing data from 10 leave samples of *C. argyrophylla*, and compared these events with those in closely related Pinaceae species and other gymnosperms (**Figure 2c**). Our analysis identified a total of 1,201 RNA editing sites in *C. argyrophylla*, with the majority (1,172) being C-to-T edits. Remarkably, *C. argyrophylla* possesses the highest number of RNA editing sites among all analyzed mitogenomes (**Figure 2c**). While a general trend links larger mitogenomes (starting around 1 Mbp) with increased editing sites (**Figure 2c**), this pattern doesn’t hold for smaller genomes (below 1 Mbp). For instance, *L. tulipifera*, despite its diminutive 0.5 Mbp genome, exhibits a striking 755 editing sites, significantly surpassing the 225 sites found in the 1 Mbp genome of *W. mirabilis*. This contrast underscores that the correlation between mitogenome size and RNA editing site abundance is not straightforward across all size ranges, potentially hinting at varying selective pressures or regulatory nuances affecting editing in smaller versus larger genomes.

### 3.5 Relationship between plastid-to-mitochondrial DNA transfers and genome size in Pinaceae

**Figure 3:**
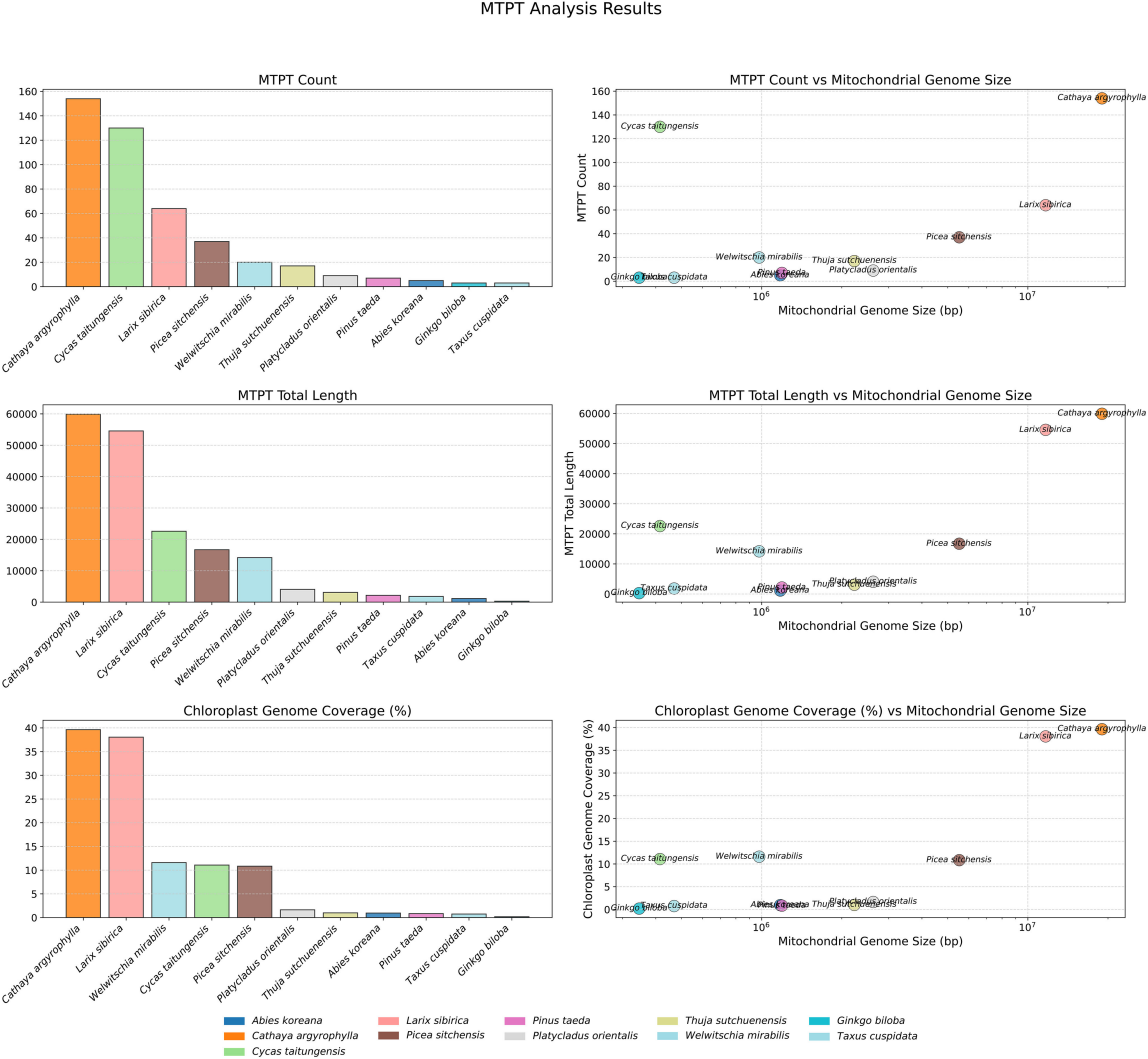
Comparative analysis of mtpts between *C. argyrophylla* and other gymnosperms.

To further compare the transfer of plastid genome sequences into the mitogenomes of large Pinaceae species and other gymnosperms, we analyzed plastid-derived fragments (mtpts) in these genomes. The results showed that the number, total length, and coverage of mtpts in the chloroplast/plastid sequences were higher in the extremely large mitogenomes of Pinaceae (*C. argyrophylla*, *L. sibirica*, and *P. sitchensis*) compared to other gymnosperms (**Figure 3**). These three species consistently ranked among the top five for all three metrics, with *C. argyrophylla* taking the top position, indicating a positive correlation between mitogenome size and the transfer of chloroplast/plastid sequences.

This study revealed a consistent trend across all three metrics: 1 million base pairs (1 Mbp) serve as a threshold for mitogenome size (**Figure 3**). In mitogenomes larger than 1 Mbp, it showed a positive correlation with the number, length, and coverage of mtpts, while this relationship did not hold in genomes smaller than 1Mbp. This suggests that mitogenomes larger than 1 Mbp might experience different mechanisms or regulatory patterns for sequence exchange with the plastid genome compared to smaller gymnosperm mitogenomes.

**Figure 4:**
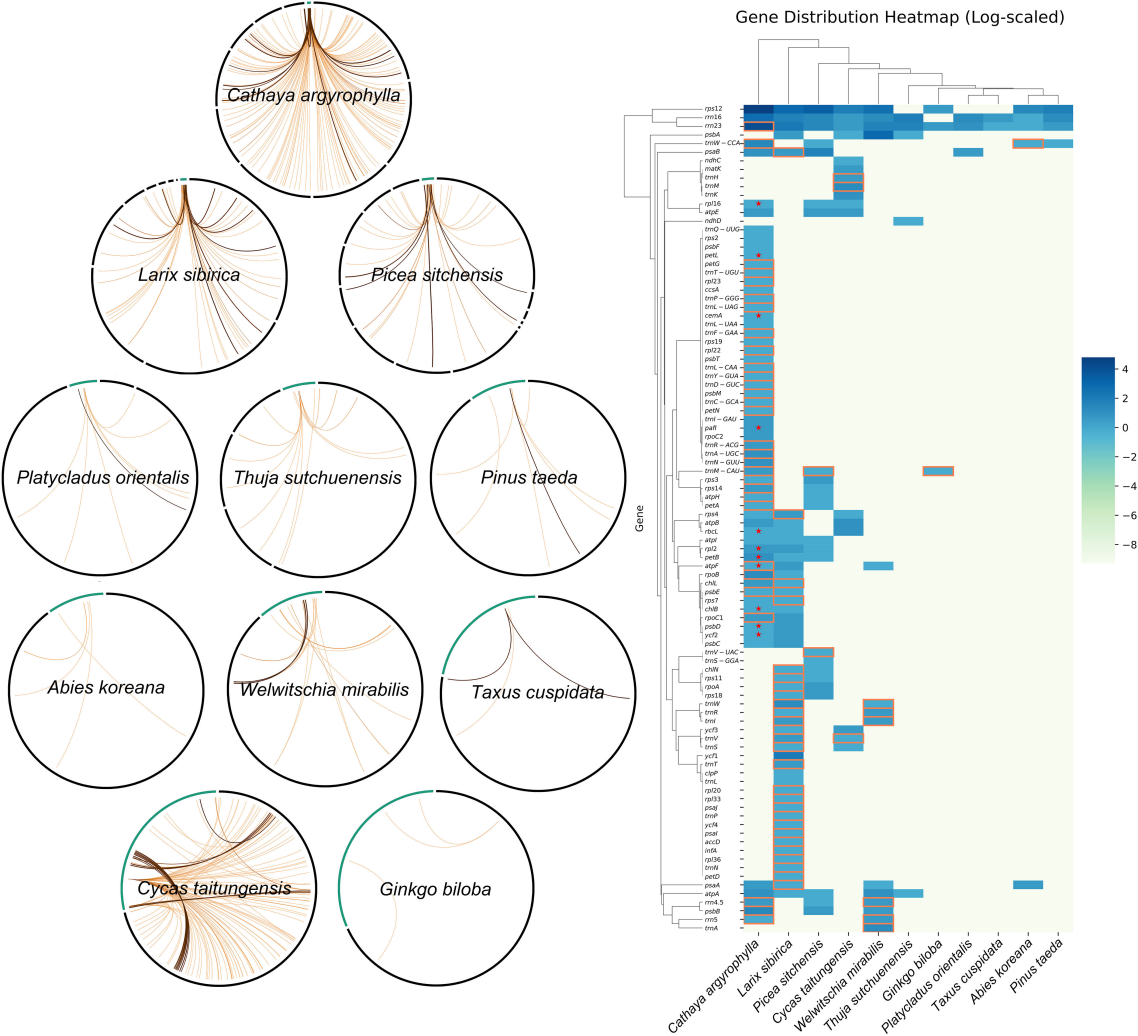
Comparison of plastid genes contained within mtpt sequences between *C. argyrophylla* and other species.

Upon closer examination, it was observed that 11 plastid genes transferred into the mitogenome of *C. argyrophylla* corresponded to previously identified polymorphic plastid genes across multiple *C. argyrophylla* populations (**Figure 4**), accounting for 57.90% of all 19 known polymorphic plastid genes. This finding suggests that these highly active polymorphic genes are more likely to be transferred into the mitogenome of *C. argyrophylla*. Additionally, a comparison between *C. argyrophylla* and *L. sibirica* revealed that only a few of the transferred genes were shared between the two species, indicating that the patterns of gene transfer between mitochondrial and plastid genomes can vary significantly between species.

### 3.6 Analysis of unique mitochondrial-nuclear DNA transfer in Pinaceae

To investigate the extent and nature of sequence exchange between the mitochondrial and nuclear genomes (NUMTs) in Pinaceae, particularly focusing on species with large mitogenomes like *C. argyrophylla*, we conducted a comparative analysis involving other gymnosperms and angiosperms. Homologous fragments between the mitogenomes of selected gymnosperms and reference nuclear genomes were identified using BLASTn. The nuclear genomes of *Larix sibirica*, *Picea sitchensis*, *Pinus taeda*, *Ginkgo biloba*, and *Cycas panzhihuaensis* (used as a proxy for *Cycas taitungensis*, whose complete nuclear genome is unavailable) served as the index databases for these comparisons.

**Figure 5:**
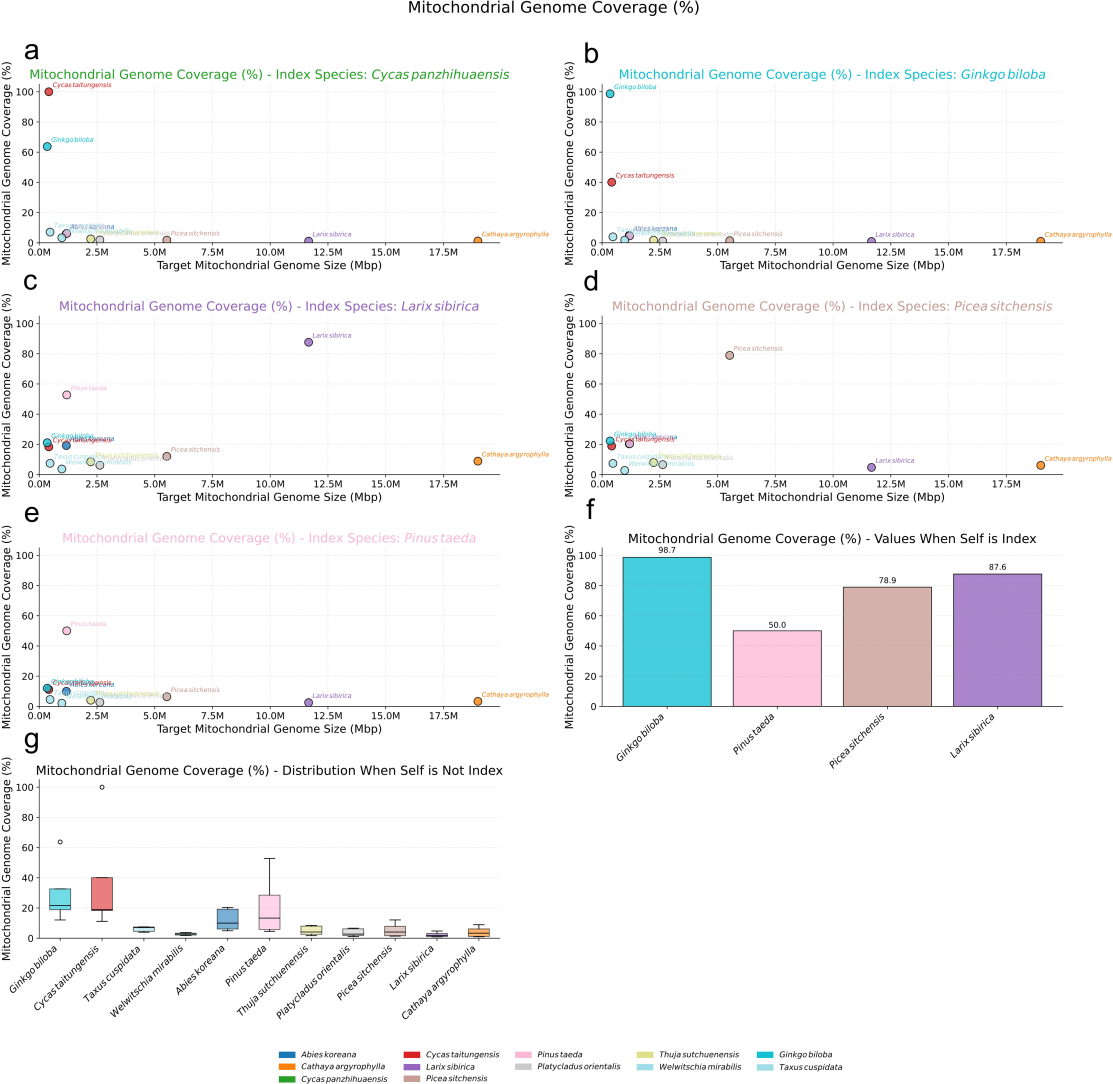
Nuclear Mitochondrial DNA Sequences (NUMTs) Analysis in Pinaceae. **(a-e)** Scatter plots illustrating the percentage of mitochondrial genome coverage (%) for various target gymnosperm mitogenomes (plotted by size on the x-axis) when aligned against specific reference nuclear genomes used as an index: **(a)** Cycas panzhihuaensis, **(b)** Ginkgo biloba, **(c)** Larix sibirica, **(d)** Picea sitchensis, and **(e)** Pinus taeda. Each colored dot represents a target mitogenome species as indicated in the bottom legend. **(f)** Bar chart showing the mitochondrial genome coverage (%) when the nuclear genome of the same species is used as the index, for the species whose nuclear genomes served as indices. **(g)** Box plots depicting the distribution of mitochondrial genome coverage (%) for each target mitogenome (labeled on x-axis) when aligned against all other (non-self) nuclear genome indices.

Our results revealed a generally low level of sequence homology, measured as the proportion of the mitogenome covered by homologous nuclear sequences, within the Pinaceae family (**Figure 5**). When utilizing the nuclear genome of the same species as the index, the mitogenome coverage varied, reaching a maximum of approximately 87.61% in *L. sibirica* (**Figures 5c, f**) but only 50.04% in *P. taeda* (**Figures 5e, f**). Notably, within Pinaceae, a positive correlation was observed between mitogenome size and this same-species NUMT coverage (**Figure 5f**): coverage increased from 50.04% in *P. taeda* (1.19 Mbp mitogenome) to 78.94% in *P. sitchensis* (5.52 Mbp) and 87.61% in *L. sibirica* (11.66 Mbp). However, when using the nuclear genome of a different species as the index, the coverage of Pinaceae mitogenomes dropped substantially. This decrease was particularly pronounced for species with extremely large mitogenomes, such as *C. argyrophylla* (18.99 Mbp) and *L. sibirica* (11.66 Mbp). Their mitogenome coverage fell below 10% when compared against the nuclear index of more distantly related gymnosperms like *C. panzhihuaensis* (**Figure 5a**) or *G. biloba* (**Figure 5b**), consistently ranking them lowest among the species tested. Even when using the nuclear index of other, more closely related Pinaceae species, the coverage for *C. argyrophylla* and *L. sibirica* mitogenomes remained relatively low, below 10%, typically within the bottom five rankings (**Figure 5g**).

Conversely, Pinaceae species with smaller mitogenomes exhibited considerably higher coverage when compared against the nuclear index of other Pinaceae species. For instance, while *P. taeda* (1.19 Mbp), *Abies koreana* (1.17 Mbp), and *P. sitchensis* (5.52 Mbp) showed less than 10% coverage against the distant *C. panzhihuaensis* (**Figure 5a**) or *G. biloba* (**Figure 5b**) nuclear index, their coverage increased significantly against other Pinaceae nuclear index genomes. Specifically, using the *L. sibirica* nuclear index (**Figure 5c**), the *P. taeda* mitogenome showed 52.75% coverage (ranking second only to *L. sibirica* itself), *A. koreana* showed 19.23% coverage (ranking fourth)(**Figure 5c**), and *P. sitchensis* showed 12.09% coverage (ranking sixth)(**Figure 5c**). Similar patterns of higher relative coverage for smaller Pinaceae mitogenomes were observed when using the *P. sitchensis* (**Figure 5d**) and *P. taeda* (**Figure 5e**) nuclear genomes as the index. This pattern may indicate greater sequence conservation in the smaller Pinaceae mitogenomes compared to the larger ones, which might harbor more diverse, non-conserved sequences. Furthermore, the trend observed with same-species comparisons suggests that larger mitogenomes might engage in more extensive or recent sequence exchange with their own nuclear genomes. These observations highlight a significant distinction in NUMT patterns between Pinaceae species with extremely large versus smaller mitogenomes.

**Figure 6:**
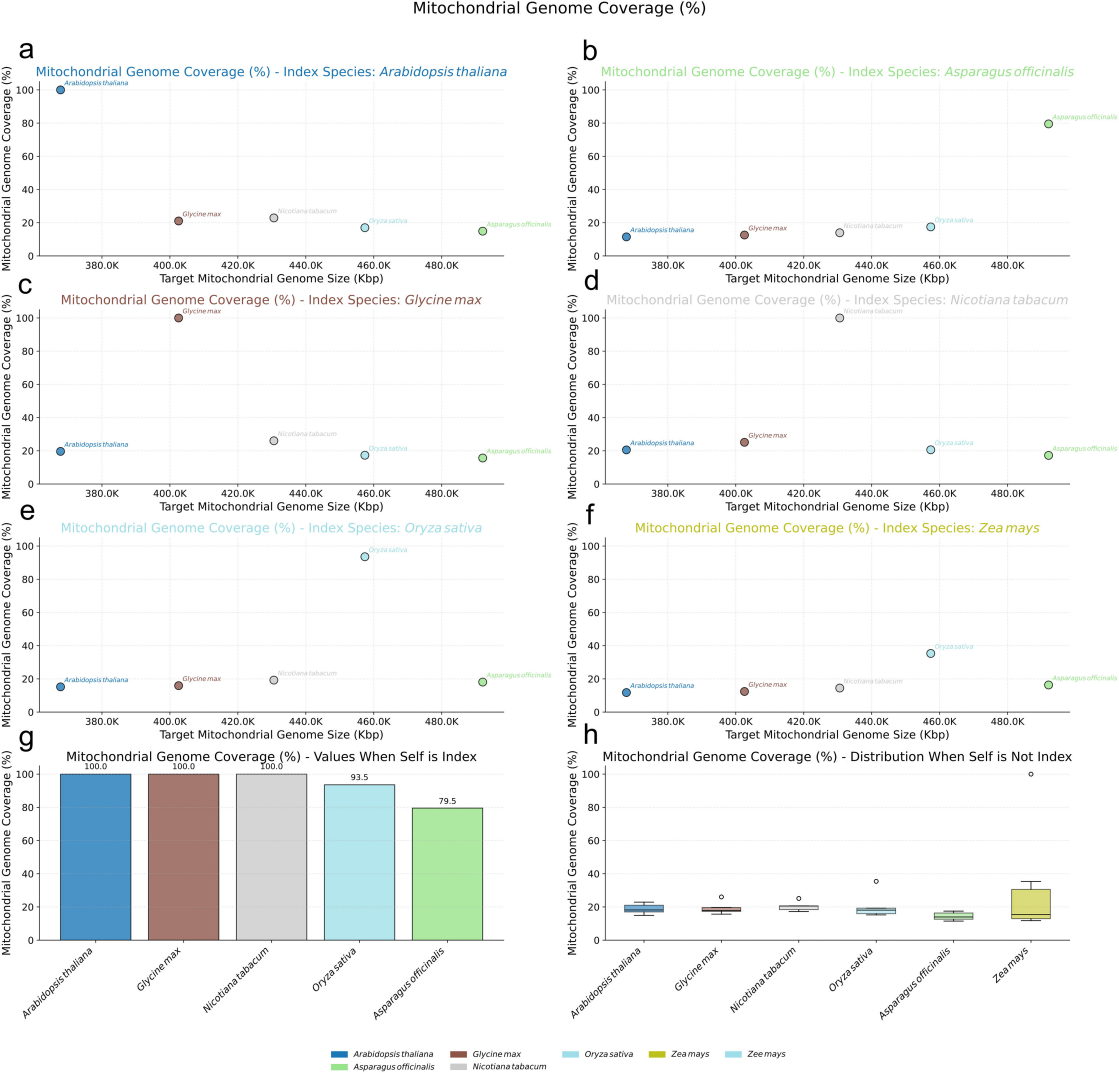
Nuclear Mitochondrial DNA Sequences (NUMTs) Analysis in Angiosperms. **(a-f)** Scatter plots illustrating the percentage of mitochondrial genome coverage (%) for various target angiosperm mitogenomes (plotted by size on the x-axis) when aligned against specific reference nuclear genomes used as an index: **(a)** Arabidopsis thaliana, **(b)** Asparagus officinalis, **(c)** Glycine max, **(d)** Nicotiana tabacum, **(e)** Oryza sativa, and **(f)** Zea mays. Each colored dot represents a target mitogenome species as indicated in the bottom legend. **(g)** Bar chart showing the mitochondrial genome coverage (%) when the nuclear genome of the same species is used as the index, for the species whose nuclear genomes served as indices. **(h)** Box plots depicting the distribution of mitochondrial genome coverage (%) for each target mitogenome (labeled on x-axis) when aligned against all other (non-self) angiosperm nuclear genome indices.

In stark contrast, both angiosperms and other gymnosperms possessing smaller mitogenomes displayed evidence of much higher mitochondrial-nuclear sequence exchange (**Figures 6a–f**). Angiosperm mitogenomes consistently showed nearly 100% coverage when compared against their own species’ nuclear index (**Figure 6g**), and maintained substantial coverage, often exceeding 20%, even when compared against the nuclear index of different angiosperm species (**Figure 6h**). A similar high-coverage pattern was observed for gymnosperms with small mitogenomes like *G. biloba* and *C. taitungensis*, whose mitogenomes exhibited over 98% coverage against their respective same-species nuclear index (**Figure 5f**) and surpassed 40% coverage against different-species gymnosperm nuclear indexes (**Figure 5g**).

These findings suggest that the mechanisms governing sequence exchange between the mitochondrial and nuclear genomes in Pinaceae species, particularly those characterized by extremely large mitogenomes, may differ significantly from those operating in angiosperms and other gymnosperms with smaller mitogenomes. This divergence could reflect complex and potentially unique regulatory pathways influencing NUMT dynamics within Pinaceae.

## 4 Discussion

**Figure 7:**
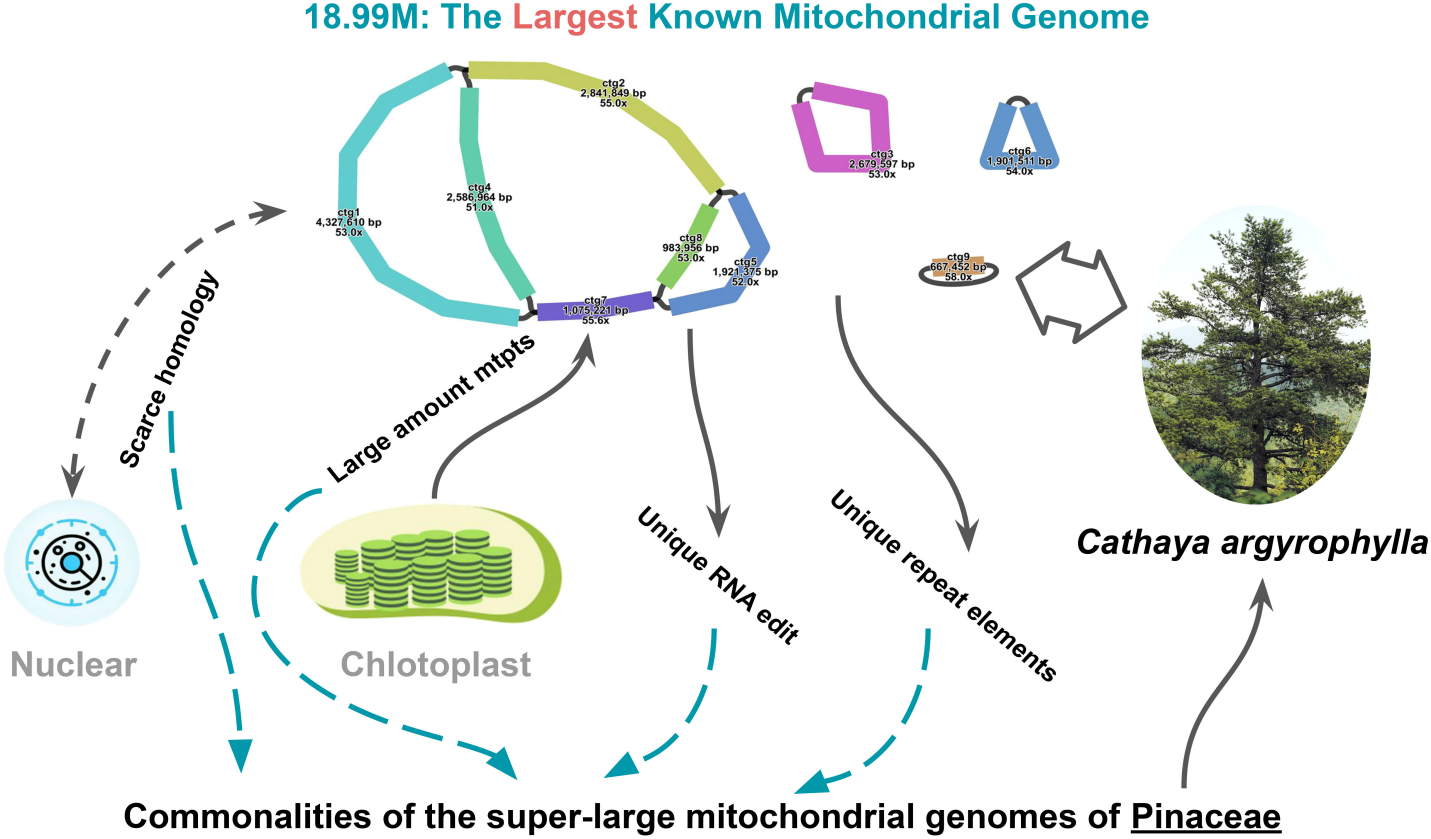
Visual summary of the 18.99 Mb Cathaya argyrophylla mitogenome, illustrating contributing factors to its unprecedented size and shared characteristics of Pinaceae super-large mitogenomes.

This study conducted a comprehensive analysis of the mitogenome of *C. argyrophylla*, uncovering its unique genomic features and comparing them with other Pinaceae species and gymnosperms. Through the investigation of repeat sequences, transposon activity, RNA editing events, chloroplast-derived fragments (mtpts), and the sequence homology between mitochondrial and nuclear genomes, we explored the common mechanisms and significance of extremely large mitogenomes in Pinaceae (**Figure 7**).

Previous studies have consistently reported the large size and structural complexity of Pinaceae mitogenomes, as exemplified by findings in *L. sibirica* (Putintseva et al., 2020) and *P. sitchensis* (Jackman et al., 2020). In this study, we discovered that the mitogenome of *C. argyrophylla* is the largest among all Pinaceae species, with a genome size of 18.99 Mb, surpassing the previously reported largest mitogenome in *L. sibirica* (Putintseva et al., 2020), setting a new record for mitogenome size. This massive genome primarily consists of a multi-branched structure, and high-precision Nanopore sequencing and assembly revealed 6 linear contigs and 3 circular contigs. This is similar to the complex multi-contig structures found in *L. sibirica* (9 contigs) and *P. sitchensis* (13 contigs). However, compared to other extremely large mitogenomes in Pinaceae, the genome size of *C. argyrophylla* shows a significant increase, highlighting not only the shared characteristics of Pinaceae species but also the unique evolutionary features of *C. argyrophylla*.

Synteny analysis plays a crucial role in understanding genome structure and evolution. By comparing syntenic relationships between genomes of different species, we can uncover evolutionary events such as genome rearrangements and infer phylogenetic relationships (Small et al., 2020; Sloan et al., 2018; Sang et al., 2019; Kovar et al., 2018; Rawal et al., 2020). In this study, synteny analysis revealed that *C. argyrophylla* shares numerous syntenic blocks with other Pinaceae species, with the number of syntenic blocks closely correlating with phylogenetic relatedness. This indicates that the mitogenomes of Pinaceae have maintained a certain level of conservation throughout evolution. However, we also observed that the number of syntenic blocks is influenced by mitogenome size. Species with larger mitogenomes tend to share more syntenic blocks. While this could simply be a consequence of larger genomes increasing the statistical probability of detecting homologous sequences, we do not rule out the alternative possibility that genome expansion itself may facilitate sequence sharing and recombination. If the latter is the case, this finding supports the role of genome size in influencing genome structure and evolution, indicating that larger genomes may have undergone more frequent recombination and rearrangement events. Further in-depth research is required to confirm this hypothesis.

Repeat sequence analysis is a key tool for understanding genome expansion and increasing complexity. The accumulation of repeat sequences can lead to genome size enlargement and influence genome stability and functionality (Small et al., 2020; Yu et al., 2022; Chevigny et al., 2020; Dong et al., 2018; Martins et al., 2019). In this study, we found that while *C. argyrophylla* and *L. sibirica* have the highest total length of repeat sequences and the highest total length of their genomes covered by repeats, their overall repeat sequence coverage rate is not the highest. This suggests that even extremely large mitogenomes are not predominantly composed of repeat sequences. Additionally, in extremely large mitogenomes, the proportion of repeat sequences longer than 200 bp increases with genome size, a trend that is not observed in smaller genomes. This implies that extremely large mitogenomes may have unique mechanisms for the formation and accumulation of repeat sequences.

Transposon activity plays a crucial role in genome evolution and structural variation. The insertion and amplification of transposons can lead to changes in genome size and gene content (Mower, 2020; Small et al., 2020; Wang et al., 2021; Li et al., 2021). Previous studies have shown that plant mitogenomes contain relatively few transposon types and numbers, particularly the rare LINEs and LTR elements (Putintseva et al., 2020; Clifton et al., 2004; Knoop et al., 1996), and our research confirms this observation. However, our study further reveals an increase in the presence of LINEs and LTR elements in extremely large mitogenomes. We found that as mitogenome size increases, the proportion of LINEs and LTR elements also rises, suggesting that transposon activity may have played a key role in the expansion of extremely large mitogenomes. However, in contrast, this trend was not observed in smaller mitogenomes, and in some cases, an unusually high proportion of LTR elements was found (such as in *A. koreana*), indicating that different regulatory mechanisms may govern transposon activity in genomes of different sizes. Despite the potential link between extremely large mitogenome size and the increased activity of certain transposon types like LINEs and LTR elements, repeat sequences may only partially explain the massive size of large Pinaceae mitogenomes. For instance, even in *C. argyrophylla*, which has the highest mitogenome size, these elements account for only 14.78% of the genome, suggesting that other factors contribute to the substantial genome size.

RNA editing, a post-transcriptional alteration of RNA sequences, is prevalent in plant mitochondria and influences gene function, impacting processes like respiration and protein synthesis (Small et al., 2020; Bentolila et al., 2013). Our analysis revealed that *C. argyrophylla* boasts the highest number of RNA editing sites among the examined species, with the majority being C-to-U edits. While a general trend links increasing genome size with more editing sites, this relationship is not strictly linear, particularly in smaller genomes. The high number of editing sites in *C. argyrophylla*, coupled with its enormous genome size, suggests that extensive RNA editing may be a hallmark of these large mitogenomes. This increased editing could reflect a greater complexity in gene regulation and protein function required to maintain the expanded genome, or it could be a consequence of increased susceptibility to mutagenic processes within the larger genome. Further research is needed to elucidate the precise role of RNA editing in the evolution and maintenance of these exceptionally large mitogenomes, and whether the observed patterns relate to functional adaptations or are simply a byproduct of genome expansion. This heightened RNA editing activity in larger mitogenomes could potentially explain functional differences compared to smaller genomes and warrants further investigation.

The transfer mtpts is a key pathway for inter-organellar genome exchange. The integration of mtpts can enlarge mitogenomes and provide new sequences for genome recombination (Mower, 2020; Small et al., 2020; Sullivan et al., 2020; Martins et al., 2019; Xia et al., 2023). Our study shows that *C. argyrophylla*, with the largest mitogenome, has the highest number, total length, and coverage of mtpts in its mitogenome, and for species with mitogenome sizes exceeding 1 Mbp, all mtpt metrics are positively correlated with genome size. Notably, the mtpt coverage in *C. argyrophylla* and *L. sibirica* extends to approximately 40% of the chloroplast genome, far exceeding that of other species. This suggests that larger mitogenomes may be more receptive to incorporating plastid sequences, thereby promoting genome expansion and increased complexity. Furthermore, a significant proportion of the transferred plastid genes were previously identified as polymorphic chloroplast genes in *C. argyrophylla* (Huang et al., 2022), confirming their activity and laying a foundation for further functional studies of these genes.

The exchange between mitochondrial and nuclear genomes is crucial for understanding genome evolution and organelle-nucleus interactions. In many plants, mitochondrial DNA fragments can be transferred to the nuclear genome, forming nuclear mitochondrial DNA sequences (NUMTs) that influence the structure and function of the nuclear genome (Jiang et al., 2023; Wang et al., 2021; Richly and Leister, 2004; Hazkani-Covo et al., 2010). However, we found that the sequence homology between the mitochondrial and nuclear genomes in Pinaceae species is relatively low, contrasting with findings in angiosperms and other gymnosperms. There are two possible explanations for this phenomenon: (1) Pinaceae species may have reduced mitochondrial-nuclear genome exchange, resulting in lower homology coverage; (2) The high variability and complexity of mitogenome sequences in Pinaceae could complicate sequence comparisons, leading to lower coverage when the nuclear genome of the same species is used as the index. Additionally, as the mitogenome size increases in Pinaceae species, the coverage of homologous fragments within the mitogenome itself also increases when using the species’ own nuclear genome as a index. This may indicate that the expansion of mitogenomes could enhance the potential or activity of sequence exchange with the nuclear genome. However, when using the nuclear genome of a different species as the index, the coverage of homologous fragments tends to be lower for larger Pinaceae mitogenomes, particularly when the index species is phylogenetically distant. This suggests that these larger mitogenomes may have undergone more numerous or complex evolutionary changes, potentially resulting in a smaller proportion of sequences conserved across species. Further research is required to clearly define the relationship between mitogenome size and the retention of conserved sequences within Pinaceae.

Based on the results, we hypothesize that the formation of large mitogenomes in Pinaceae species is likely the result of multiple contributing factors. While the increase in repeat sequences and transposon activity may have facilitated genome expansion, they are not the primary driving forces. Instead, the extensive incorporation of plastid sequences appears to play a more critical role in genome enlargement. Additionally, the increased number of RNA editing events may be linked to the heightened complexity of the genome, reflecting the functional regulatory demands of large mitogenomes. Furthermore, the relatively limited exchange between the mitochondrial and nuclear genomes in Pinaceae suggests that unique mechanisms may govern their interaction. These factors exhibit different patterns in large mitogenomes compared to smaller ones, indicating that Pinaceae species might possess a distinct evolutionary mechanism for mitogenome development. These findings are important for understanding the structure, function, and evolution of plant mitogenomes. The uniqueness of large Pinaceae mitogenomes suggests the presence of specialized regulatory mechanisms that could influence genome stability, energy metabolism, and adaptive evolution. Future research should further investigate these mechanisms, particularly the roles of plastid sequence transfer and transposon activity in genome expansion, as well as the processes governing sequence exchange between large mitogenomes and the nuclear genome.

It is important to acknowledge several limitations in this study, primarily concerning the number and taxonomic breadth of the species included in the comparative analyses. Given the inherent high variability and structural complexity known for plant mitogenomes, drawing definitive, family-wide conclusions based on the currently available, relatively limited set of fully sequenced Pinaceae mitogenomes presents challenges. The availability of complete mitogenome sequences for Pinaceae, especially representing diverse lineages and genome sizes, remains somewhat scarce. Therefore, while our analysis provides valuable initial insights into the unique features of the exceptionally large *C. argyrophylla* mitogenome and highlights potential trends associated with mitogenome expansion in Pinaceae by comparing it with selected gymnosperms, the findings should be interpreted with caution. Future investigations incorporating a larger and potentially more phylogenetically constrained set of Pinaceae mitogenomes are essential to validate the observed patterns, elucidate the generality of these features across the family, and provide a more robust understanding of the evolutionary dynamics driving mitogenome expansion in this group.

## 5 Conclusion

In conclusion, this study not only reveals an unprecedented size for the mitogenome of *C. argyrophylla* but also underscores the complex interplay of evolutionary factors that may have contributed to this expansion. By comparing multiple Pinaceae species, we found that plastid-to-mitochondrial sequence transfers, repeat sequences, transposon activity, and abundant RNA editing collectively shape the unique features of these exceptionally large mitogenomes. The relatively limited exchange with the nuclear genome further points to distinctive regulatory mechanisms in Pinaceae. These insights deepen our understanding of plant mitogenome evolution. Future studies should integrate functional genomics and molecular biology approaches to further investigate these issues. Additionally, the conservation and utilization of *C. argyrophylla* could benefit from these foundational studies, offering key insights into genetic diversity and evolutionary adaptability.

## Data Availability

The sequencing reads used in the assembly for this study are deposited in the NCBI repository under the following identifiers: BioProject PRJNA1105731, BioSample SAMN41108760, and Sequence Read Archive (SRA) data SRR28842127 (third-generation) and SRR28842128 (second-generation).

## Supplementary material

Supplementary Figure 1 Validation of contig end connectivity using MUMmer visualization. Raw reads mapped to sequences constructed from 40 kb contig ends joined by a 20 kb N-spacer show alignments spanning the junctions, confirming linkage.

Supplementary Figure 2 Read coverage across the assembled mitochondrial contigs using 200 Gb of *Cathaya argyrophylla* second-generation sequencing data.
