# Supplementary figures and images for: Super-large record-breaking mitochondrial genome of Cathaya argyrophylla in Pinaceae
Source: Front Plant Sci. 2025 Jun 19;16:1556332. doi: 10.3389/fpls.2025.1556332 (PMC12222119; doi:10.3389/fpls.2025.1556332)

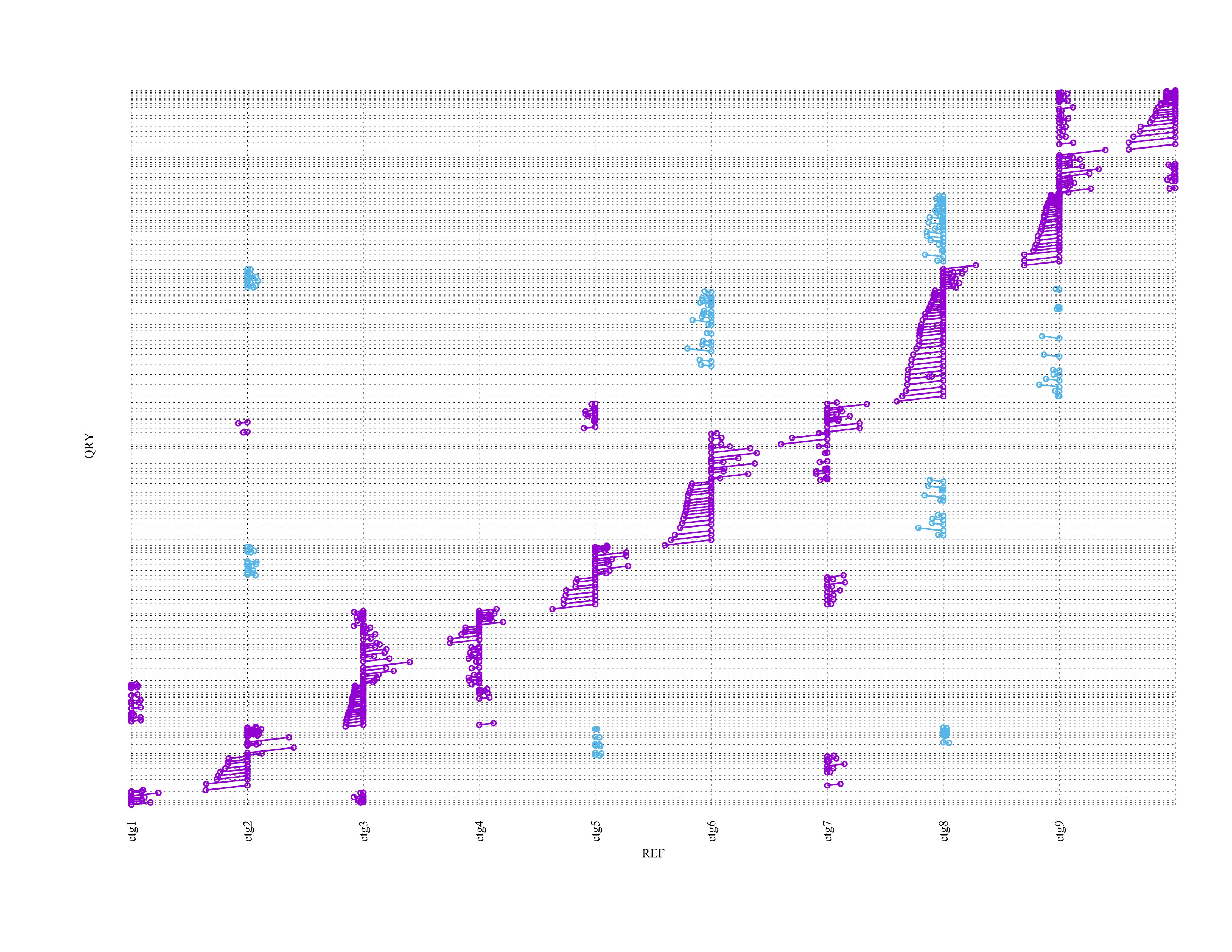

Supplement: Supplementary Figure 1 — Validation of contig end connectivity using MUMmer visualization. Raw reads mapped to sequences constructed from 40 kb contig ends joined by a 20 kb N-spacer show alignments spanning the junctions, confirming linkage. [file Image1.jpeg]

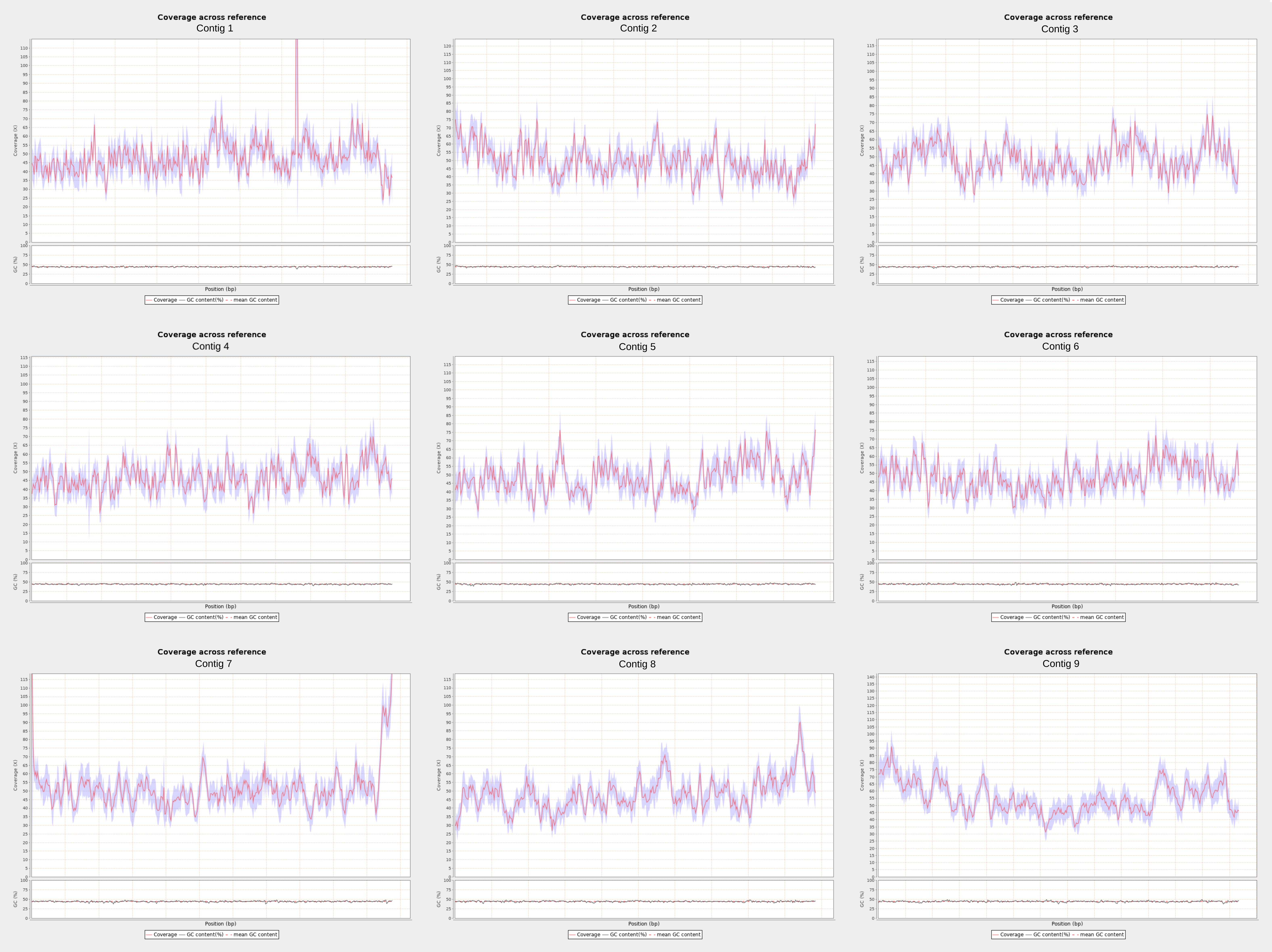

Supplement: Supplementary Figure 2 — Read coverage across the assembled mitochondrial contigs using 200 Gb of Cathaya argyrophylla second-generation sequencing data. [file Image2.jpeg]
